# Treatment of Oroantral Communication and Fistulas with the Use of Blood-Derived Platelet-Rich Preparations Rich in Growth Factors: A Systematic Review

**DOI:** 10.3390/ijms252111507

**Published:** 2024-10-26

**Authors:** Paulina Adamska, Magdalena Kaczoruk-Wieremczuk, Dorota Pylińska-Dąbrowska, Marcin Stasiak, Michał Bartmański, Adam Zedler, Michał Studniarek

**Affiliations:** 1 Division of Oral Surgery, Faculty of Medicine, Medical University of Gdańsk, 7 Dębinki Street, 80-210 Gdańsk, Poland; adam.zedler@gumed.edu.pl; 2 Individual Specialist Oral Surgery Practice Magdalena Kaczoruk-Wieremczuk, 41/31 Władysława Wysockiego Street, 17-100 Bielsk Podlaski, Poland; magdalena_kaczoruk@o2.pl; 3 Department of Dental Prosthetics, Faculty of Medicine, Medical University of Gdańsk, 18 Orzeszkowej Street, 80-204 Gdańsk, Poland; dorota.pylinska-dabrowska@gumed.edu.pl; 4 Division of Orthodontics, Faculty of Medicine, Medical University of Gdańsk, Aleja Zwycięstwa 42c, 80-210 Gdańsk, Poland; marcin.stasiak@gumed.edu.pl; 5 Institute of Manufacturing and Materials Technology, Faculty of Mechanical Engineering and Ship Technology, Gdańsk University of Technology, 11/12 Gabriela Narutowicza Street, 80-233 Gdańsk, Poland; michal.bartmanski@pg.edu.pl; 6 Department of Radiology, Faculty of Medicine, Medical University of Gdańsk, 17 Smoluchowskiego Street, 80-210 Gdańsk, Poland; michal.studniarek@gumed.edu.pl

**Keywords:** autografts, growth factor, platelet-rich fibrin, platelet-rich plasma, bone regeneration, oroantral fistula

## Abstract

The formation of an oroantral communication (OAC) or fistula (OAF) is a rare complication resulting from the presence of processes in the lateral parts of the maxilla or for iatrogenic reasons. The most common causes of OAC or OAF are tooth extraction with periapical lesions. The aims of this systematic review were to assess the use of platelet-derived preparations rich in growth factors in the treatment of OAC or OAF, to determine the success of treating the communication or fistula, as well as impact on postoperative complications and the course of healing. The study was performed following PRISMA guidelines (PROSPERO: CRD42024570758). The inclusion criteria were as follows: at least ten patients, the presence of oroantral communication or oroantral fistula, treatment with platelet-derived preparations rich in growth factors, and information regarding the response to treatment. In order to find relevant studies, international databases, including PubMed, Google Scholar, Web of Science Core Collection, MDPI, Wiley, and Cochrane Library were searched. The last search was performed on 31 August 2024. Seven articles were included in the systematic review. In total, platelet-derived preparations rich in growth factors were used in 164 patients. Only studies in which OAC was treated and with platelet-rich fibrin (PRF) met the inclusion criteria. Only PRF was used as the sole treatment method in three studies. When OAC is greater than 5 mm, platelet-derived preparations rich in growth factors should be considered adjuncts to treatment, not the sole treatment method. The success rate of OAC treatment was 90–100%. The use of blood products to close OAC may be an effective therapeutic alternative. Proper patient qualification and the use of an appropriate protocol are crucial. There is a need for future well-designed case-control or cohort studies as well as randomized controlled trials to provide the required level of evidence.

## 1. Introduction

The formation of an oroantral communication (OAC) or fistula (OAF) is a rare complication. OAC is defined as the communication between the oral cavity and the maxillary sinus and occurs within 12 to 24 h of the incident. In the case of OAF, the time from the incident exceeds 12 to 24 h, resulting in the formation of a fistula. The frequency of OACs is 0.31–4%, and OAFs is 0.2%. Complications arise due to processes in the lateral parts of the maxilla or for iatrogenic reasons. An anatomically developed alveolar recess and wide root spacing increase the risk of OACs or OAFs. These conditions most commonly occur following tooth extraction in the lateral part of the maxilla. According to various authors, OAC or OAF occurs primarily after the extraction of the first or second molar. Complications also occur during the extraction of wisdom teeth, premolars, and, more rarely, canines. This risk is increased by the presence of periapical lesions or the inflammation of the maxillary sinus mucosal membrane, where the lower wall of the maxillary sinus has been compromised. In the case of tooth extraction, factors such as the experience of the oral surgeon, the method of root separation, a minimally invasive approach during the procedure, the use of atraumatic instruments, and the use of low forces are very important. The use of minimally invasive techniques significantly reduces the occurrence of OAC/OAF or results in a very small diameter when they occur. This makes OAC/OAF closure methods gentler, more predictable, and associated with fewer complaints reported by patients. The lack of root separation or excessive forces during the procedure increases the risk of these complications. The oral surgeon must recognize such complications and provide immediate treatment [1,2,3,4,5,6,7,8,9].

Post-treatment recommendations and compliance with them by patients after the procedure are essential. Post-procedure recommendations include taking painkillers and medications that reduce the number of secretions in the nasal cavity and paranasal sinuses (e.g., pseudoephedrine), nasal drops with, e.g., xylometazoline, not blowing nose, not drinking through a straw, a moderate lifestyle, and refraining from flying or diving. The regimen of recommendations applies to the period up to 2 weeks following an OAC/OAF. Inadequate adherence to the recommendations may result in the development of an OAC/OAF in the later stage and, consequently, inflammation in the maxillary sinus [1,2,3,4,5,6,7,8,9].

Other factors that may cause OAC/OAF include implant placement in the lateral part of the maxilla (too low primary stability with an inadequate amount of bone in the vertical dimension and without predictable bone augmentation using sinus lift techniques), implant explantation, complications from sinus lift surgery, as a result of uncontrolled migration of the implant or biomaterial into the maxillary sinus, failed zygomatic or pterygoid implantation, inflammation of the bone caused by overheating of the bone during tooth extraction or implantation, inflammation of the bone induced by radiotherapy or medication osteonecrosis of the jaw, maxillary fracture or trauma, exposure of fixation material, after orthognathic surgery, infected temporary anchorage device, or after oncological procedures [1,2,3,4,5,6,7,8,9].

The main techniques for OAC/OAF closure are surgical and include: buccal advanced flap (BAD—Wassmund–Borusiewicz method), palatal flap with or without palatal artery, pedicle connective tissue graft, free gingival graft, the Bichat’s fat pad (BFP), autogenous distant flaps (tongue or temporalis muscle flap, using cartilage from the nose or ear), the application of bone block grafts taken extraorally (from hip, fibula, ribs, tibia, skull bones, iliac plate) or intraorally (from the mental area, retromolar triangle, external oblique line or edentulous process), tooth autotransplantation, bone blocks from allogeneic and xenogeneic bone banks, synthetic materials and metals, platelet-derived preparations rich in growth factors, prosthetic dentures or using low-level laser (light) therapy [1,9,10,11].

Currently, oral and maxillofacial surgery increasingly focuses on minimizing the invasiveness of procedures to improve patient outcomes and reduce recovery times. Traditional surgical techniques, such as the use of local flaps, while effective, are often associated with significant invasiveness, leading to post-operative complications, including swelling, pain, bruising, and prolonged healing times. In response to these challenges, a promising area of research is the application of platelet-derived preparations rich in growth factors, such as platelet-rich plasma (PRP) and plasma-rich in growth factor (PRGF). These autologous treatments, derived from the patient’s blood, are biocompatible and bioconsistent, significantly reducing the risk of allergic or immunological reactions. Platelet-derived preparations rich in growth factors also enhance tissue regeneration by promoting angiogenesis and reducing inflammation, making them an innovative and less invasive alternative to conventional surgical methods. This direction holds great potential for improving clinical outcomes and patient comfort in oral and maxillofacial procedures, as supported by emerging research findings [1,4,5,6,7,12,13,14,15,16,17,18,19,20,21,22,23,24].

In this paper, the authors will focus on the use of growth factor methods in OAC/OAF closure procedures. The aims of this systematic review were to assess the use of platelet-derived preparations rich in growth factors in the treatment of OAC or OAF, to determine the success of treating the oroantral communication or fistula, as well as impact on postoperative complications and the course of healing.

## 2. Material and Methods

PRISMA guidelines (Preferred Reporting Items for Systematic Reviews and Meta-Analyses) were used in this study [25]. The study protocol was registered in the PROSPERO (Prospective Register of Systematic Reviews, CRD42024570758). Additionally, a narrative review was conducted to discuss the issues further.

### 2.1. Search Criteria

#### 2.1.1. Inclusion Criteria

As a template to formulate a clinical question, the PICO (P—population, I—Intervention or exposure, C—Comparison, O—Outcome) was used. The study characteristics are exposed below.

P. At least ten patients,
I. Oroantral communication or oroantral fistula present, and treatment with platelet-derived preparations rich in growth factors,
C. Not required,
O. Oroantral communication or oroantral fistula healing success.

The articles selected for the analysis were in English. Only human studies were enrolled in the review process.

#### 2.1.2. Exclusion Criteria

Publications were excluded from the analysis if they did not meet the PICO criteria, were not in English, were not conducted on humans, or did not involve treatment with platelet-derived preparations rich in growth factors. 

### 2.2. Data Collection

To find relevant studies, international databases, including PubMed, Google Scholar, the Web of Science Core Collection, MDPI, Wiley, and the Cochrane Library, were searched. We analyzed the scientific literature concerning OAC or OAF available from 2007 to 31 August 2024. The search was conducted using PubMed, Scopus, and Google Scholar web databases. The search terms used were: ‘platelet-rich fibrin’, ‘platelet-rich plasma’, ‘PRF’, ‘PRP’, ‘PRGF’, ‘concentrated growth factors’, ‘growth factors’, ‘platelet-derived preparations rich in growth factors’, ‘CGF’, ‘A-PRF’, ‘A-PRF+’, ‘I-PRF’, ‘T-PRF’, ‘autologous fibrin glue’, ‘AFG’, ‘oroantral communication’, ‘oroantral fistula’, ‘OAC’, and ‘OAF’. References were imported into the Mendeley manager. The first stage involved screening titles and abstracts, followed by a second stage where full texts were assessed for eligibility.

Subsequently, the first author analyzed the publications and critically assessed the articles. In cases of uncertainty, the analysis was completed by a second author. The risk of bias was analyzed by the first and fourth authors. The following information was obtained from each manuscript: author and year of study, country, patient characteristics (number of cases, age), aim, OAC or OAF presence, localization, type of platelet-derived preparations rich in growth factors, platelet-derived concentrates preparation type, growth factor centrifugation method, platelet-derived growth factor preparation method, closure technique, observation period, methods of assessing the success of treatment, level of OAC or OAF closure success, additional features and conclusions. Duplicates, letters to the editor, and works of low scientific value or containing irrelevant information were removed from the analysis.

### 2.3. Quality Assessment

To assess the quality of studies with higher quality of evidence, only studies with a study group greater than or equal to ten patients were selected for bias analysis. In this article, the risk of bias was assessed using the Newcastle–Ottawa Scale (NOS). The NOS scale assesses (1) the study selection, (2) comparability, and (3) exposure [26]. These procedures were performed by the first and fourth authors. In case of disagreement, a consensus reading was made. 

## 3. Results

### 3.1. Literature Search

**Figure 1:**
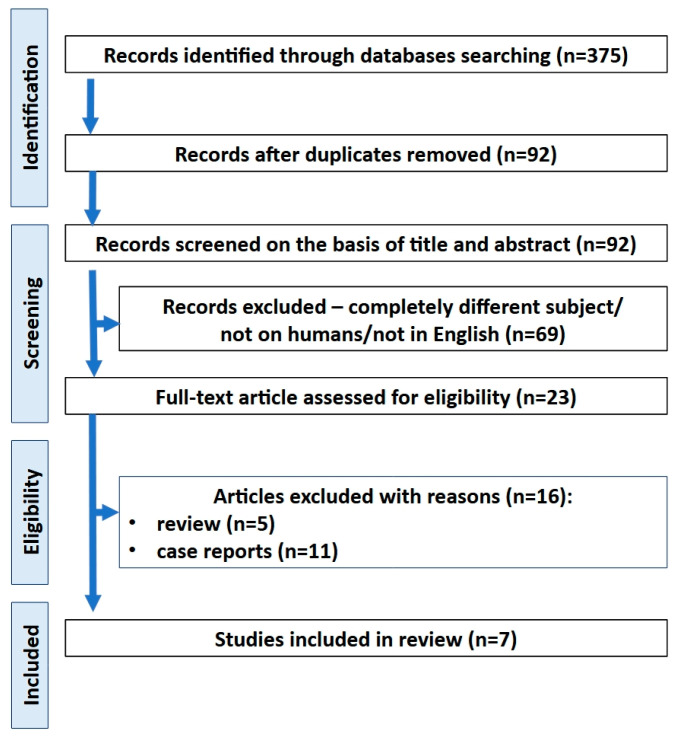
PRISMA flow diagram depicting the process followed for the selection of the studies.

Platelet-derived preparations rich in growth factors can be a promising technique for closing OAC or OAF. They stimulate healing and angiogenesis and reduce the postoperative reaction, pain, and inflammatory complications. The first reports on this subject have already been described. In the first step of selection, 375 references were identified. Ninety-two records were selected after the exclusion of duplicates. To date, only seven studies have investigated the use of PRF to close oroantral communications (Table 1 and Figure 1) [5,7,13,14,19,22,23]. No studies describing the treatment of OAF using platelet-derived platelet-rich growth factors that met the inclusion criteria were found. The number of studies was limited and consisted of descriptive studies with low evidence. Due to the low number of reports meeting the inclusion criterion of ten cases and the lack of description of OAF treatment using this method, and only seven studies about OAC, the case descriptions are included in the Supplementary Table S1 [1,4,6,9,10,12,13,14,15,20,21,24].

The first paper that met the inclusion criteria was published in 2016 [23]. There were four studies from Turkey [5,7,19,22], two from Poland [13,23], and one from Austria [14].

### 3.2. Study Characteristic

In total, platelet-derived preparations rich in growth factors were used in 164 patients [5,7,13,14,19,22,23]. All studies focused on the closure of oroantral communications. The largest study group consisted of 50 patients [14]. Six studies used clinical examination to assess the presence of OAC [5,7,13,14,19,23], and four additionally used radiological examination [5,13,14,23]. The primary cause of OAC was tooth extraction [14,19], particularly the first molar, followed by the second molar. Among the platelet-derived preparations rich in growth factors, platelet-rich fibrin (PRF) was used in six studies [5,7,14,19,22,23], and advanced platelet-rich fibrin (A-PRF) in one study [13]. The main method after centrifugation was to apply the concentrate to the prepared wound as a clot [5,7,13,14,22] and then as a membrane [19,23].

In three studies, the platelet-rich fibrin was used as the sole treatment method [7,13,22], and in four studies, the combined PRF and free tension flat/BAF/BFP [5,14,19,23]. The results of treatments with platelet-derived preparations rich in growth factors were very good, and all patients had good healing in follow-up. The shortest follow-up was 14 days [13], and the longest was six months [23]. An observation period of less than two weeks would be insufficient. The effectiveness of treatment was assessed primarily clinically and also by radiological diagnostics. Treatment success in studies ranged from 90 to 100%. Treatment success was defined as the complete healing of the OAC. Most authors also reported that in addition to successful healing after OAC, there was a reduction in post-procedure complications such as pain and swelling.

### 3.3. Risk of Bias

#### Newcastle–Ottawa Scale (NOS)

It was considered that two studies were of good quality and five were of low quality. The risk of bias assessment using the NOS is described in Table 2.

## 4. Discussion

Wound healing is a complex biological process that occurs after tissue injury, involving various cell types regulated by multiple growth factors and cytokines. It is a highly organized cascade of events that includes four phases: hemostasis, inflammation, proliferation, and remodeling [27]. Angiogenesis plays an important role in wound healing. Importantly, blood vessels not only deliver oxygen and nutrients but also provide instructive regulatory signals to the surrounding cells in the local tissues and thereby play key roles in organ morphogenesis and regeneration. Angiogenesis is facilitated through the interaction of various angiogenic factors and their receptors. Platelets also store and release many bioactive angiogenic factors [28]. Growth factors play a main role in cell migration, cell proliferation, and angiogenesis in the tissue regeneration phase. These growth factors are mainly located in blood plasma and platelets. As a result, platelet aggregates have been widely used to accelerate tissue regeneration and repair in dental and medical fields [29]. Growth factors are endogenous signaling molecules that regulate cellular responses required for wound healing processes such as migration, proliferation, and differentiation [30]. Growth factors play a crucial role in tissue regeneration by directing the cell fate and allowing the formation of tissues [31]. Tissue engineering aims to repair, restore, and regenerate lost or damaged tissues by using biomaterials, cells, mechanical forces, and factors (chemical and biological) alone or in combination. Growth factors are routinely used in the tissue engineering approach to expedite the regeneration process [32].

Three generations of platelet concentrates are known. First-generation platelet concentrates are platelet-rich plasma (PRP) and plasma-rich in growth factor (PRGF). Second-generation platelets aggregation are platelet-rich fibrins (PRF); third-generation are advanced platelet-rich fibrin (A-PRF), advanced platelet-rich fibrin plus (A-PRF+), injectable platelet-rich fibrin (I-PRF), concentrated growth factor (CGF), titanium platelet-rich fibrin (T-PRF) and autologous fibrin glue (AFG). Several techniques for platelet concentrates are available. Each method leads to a different product with distinct biological properties and potential uses [29,33,34,35,36,37,38,39].

Generally, platelet-derived preparations rich in growth factors are autologous materials consisting solely of the patient’s own blood elements, without anticoagulants and thrombin obtained from an animal source or other agents. As a consequence of the above, they are biocompatible and bioconsistent, do not cause allergies, and there is no risk of cross-infection. The primary limitation is taking blood from the patient, which may cause bruising and pain at the injection site. Blood is most often collected from the basilic or cephalic vein. The skin must be disinfected before the procedure. Most often, an even number of tubes are collected, and the centrifugation process is started as soon as possible. Each blood product has different centrifugation parameters [40,41].

Platelet-rich plasma is an autogenous preparation that stimulates angiogenesis by means of a release of growth factors, such as PDGF (platelet-derived growth factor), IGF (insulin-like growth factor), TGF-β (transforming growth factor beta), VEGF (vascular endothelial growth factor), FGF (fibroblast growth factor) and EGF (epithelial growth factor). These bioactive proteins play pivotal roles in stimulating cell migration, proliferation, and differentiation, as well as in forming new blood vessels and collagen. Together, these factors create a favorable environment for tissue regeneration, making PRP a valuable therapeutic tool. PRP is produced by centrifugating peripheral blood at 3200 rpm for 4 min. It is not very stable, which is why modified blood products are more important. In oral and maxillofacial surgery, PRP is used in bone regeneration, the reconstruction of the alveolar process, and periodontal tissue surgery due to its properties that stimulate angiogenesis and tissue regeneration [33,34,42,43]. PRP was also utilized as a growth factor used in the treatment of OAF. This was one case report; therefore, it is not further analyzed in this study ([24,42], Supplementary Table S1).

Plasma rich in growth factors differs from PRP in preparation. Instead of animal-derived thrombin, calcium is used to clot. This makes it more stable. PRP is produced by centrifugating peripheral blood at 1850 rpm for 8 min. PRGF contains growth factors, including PDGF, TGF-β, insulin-like growth factor (IGF), VEGF, FGF, and hepatocyte growth factors (HGF). In oral and maxillofacial surgery, PGGF is used in guided bone regeneration, socket preservation after tooth extraction, or maxillary sinus floor augmentation [34,35,36,37]. PRGF has not been used in the treatment of OAC and OAF.

Our systematic review identified that platelet-rich fibrin (in most studies) and advanced platelet-rich fibrin preparations were used for the closure of OAC. The obtained blood preparations can be used during the procedure as a clot, plug, or membrane (Table 1). Therefore, in the following sections, PRF and A-PRF will be presented in more detail. Other third-generation preparations have not been used in the treatment of OAC/OAF.

Platelet-rich fibrin consists of a three-dimensional fibrin matrix rich in platelets and leukocytes containing cytokines, stem cells, and growth factors. This biodegradable scaffold promotes microvascularization and encourages the migration of epithelial cells to its surface [29]. Platelets contained in PRF stimulate angiogenesis by means of a release of growth factors, such as PD-EGF (platelet-derived epidermal growth factor), PDGF, VEGF, FGF, or TGF. PRF is produced by centrifugating peripheral blood at 2700 rpm for 12 min [33,44,45]. Additionally, in advanced platelet-rich fibrin (A-PRF), substantial amounts of PDGF-AB, TGFβ-1 (tumor necrosis factor β-1), and TSP-1 (thrombospondin-1) can be found. The presence of leukocytes in A-PRF guarantees a higher content of pro- and anti-inflammatory mediators, TNFα (tumor necrosis factor α), and interleukins (IL-1β, IL-6, and IL-4). Leukocytes also release VEGF and TGFβ-1 [44,45,46,47,48]. PRF is produced by centrifugating peripheral blood at 1500 rpm for 14 min [33,44,45]. Choukroun’s protocol includes a higher concentration of monocytes, which are believed to release bone morphogenetic proteins BMP-2 and BMP-7 (bone morphogenic factors 2 and 7), as well as VEGF [45]. PDGF plays a vital role in each phase of the wound-healing process. PDGF is released from degranulating platelets following an injury and initiates an inflammatory response by stimulating the mitogenicity and chemotactic abilities of cells such as neutrophils, macrophages, fibroblasts, and smooth muscle cells at the site of the wound. The VEGF family consists of several members. VEGF-A initiates the process of wound healing by promoting biological processes and events such as early angiogenesis and, especially, the migration of endothelial cells [27]. FGFs make up a large family of polypeptide growth factors—there are 22 members of the FGF family [49]. These factors play crucial roles in wound healing. The sources of FGFs are keratinocytes, fibroblasts, endothelial cells, smooth muscle cells, chondrocytes, and mast cells. FGF-2 is responsible for granulation tissue formation, re-epithelialization, and remodeling [27]. Activation of the TGF-β R1 signaling pathway drives the expression of strongly regulated genes in gingival fibroblasts, including the growth factor BMP2 and, consequently, ID1 and ID3, which can generate a change in the autocrine/paracrine environment of a defect site and significantly affect gingival fibroblasts [50]. Stem cells are cells that can retain prolonged self-renewal ability and can differentiate into numerous tissue types. There are different sources of stem cells, such as embryonic stem cells, induced pluripotent stem cells, bone marrow stem cells (BMSCs), and adipose-derived stem cells (ASCs) [1,27,51].

Available studies suggest that the use of platelet-derived preparations rich in growth factors (PRF, A-PRF) can be used as the sole treatment method only in the case of OAC up to 5 mm in size. However, when the diameter of OAC is greater than 5 mm, platelet-derived preparations rich in growth factors support healing and angiogenesis, and the communication must be combined with more traditional surgical techniques to achieve successful closure. In the case of extensive OAC/OAF and the use of flaps, it is very important to prepare the flaps in such a way that they completely close the communication/fistula without tension and tightly. Local flaps should be designed so that the pedicle is wider than the free end, which ensures good blood supply. For larger defects, additional methods such as buccal advanced flaps (BAF) or palatal flaps are required to provide adequate mechanical support and ensure proper closure. These combined approaches leverage the regenerative benefits of growth factors while addressing the structural challenges posed by larger defects, ultimately improving surgical outcomes and reducing the risk of complications. Failure to follow these rules can lead to flap necrosis, wound opening, and treatment failure [1,4,5,6,7,12,13,14,15,16,17,18,19,20,21,22,23,24].

Closing OACs/OAFs is not a straightforward procedure. In the case of advanced inflammatory lesions in the maxillary sinus, inflammatory exudate, the sinus should be closed. To assess the condition of the maxillary sinus before the procedure, which will involve opening the sinus or after the detection of OAFs/OACs, imaging diagnostics should be available. Inflammatory lesions and fistulas are best visible in magnetic resonance imaging (MRI) with a contrast agent, then in computed tomography (CT), in some ultrasound techniques, in a cone beam computed tomography (CBCT), and sometimes in an orthopantomographic (OPG) examination [1,52,53,54,55,56,57]. The standard imaging evaluation of soft tissues and their abnormalities is MRI and ultrasound. MRI is widely used in the diagnosis of genitourinary or anal fistulas [58,59,60]. However, the examination of choice in oral and maxillofacial surgery is CBCT. Soft tissues are not visible in CBCT. When additional methods are used, e.g., soft tissue retraction maneuver, additional information about the condition of soft tissues can be obtained. Sometimes, hypertrophy and inflammation in the maxillary sinus are also observed [1,52,53,54,55,56,57]. Symptoms of inflammation include patient-reported congestion, nasal root pain (especially in the morning), finding purulent content draining down the throat, the presence of exudate by OAF/OAC, or the discoloration of potassium permanganate during sinus rinsing. Inflammation should be treated before proceeding with the OAC/OAF closure. Treatment involves the use of drugs from the group of antibiotics, non-steroidal anti-inflammatory drugs, reducing exudate—pseudoephedrine, xylometazoline—in the case of acute inflammation, or local and general steroids—in the case of chronic inflammation [1,52,61]. A maxillary sinus free of inflammation or with well-managed chronic inflammation is also a guarantee of the supply and success of OAC/OAF treatment.

After the OAC/OAF closure procedure, the patient is required to maintain a moderate lifestyle and avoid increasing pressure in the collateral sinuses, such as blowing their nose, flying, or heavy lifting, and to take medications that reduce maxillary sinus effusion and anti-inflammatory drugs to manage pain and swelling. [1,2,3,4,5,6,7,8,9] The time of observation after the procedure is also important. In the analyzed studies, the minimum time was 14 days [13], and the longest was six months [23]. The minimum observation time should be 14 days to assess soft tissue healing and the tightness of the treatment. A full assessment of soft and hard tissue healing should be reassessed after six months. This is related to the physiological process of bone healing. Regular follow-up ensures that complications are identified early and healing progresses as expected.

This publication is one of the first comprehensive articles to summarize the role of platelet-derived preparations rich in growth factors in the treatment of oroantral communications and fistulas. The systematic review shows the limited number of studies on this topic. A critical summary of current scientific reports has been presented. The limitations of this study are the access to a small number of studies, their descriptive nature, small study groups, and different methods of diagnosis. In the reviewed articles, different imaging techniques were used to assess the presence of complications and the condition of the maxillary sinus (orthopantomographic examination, CBCT, or Wathers X-ray), but there were also studies where the condition of the maxillary sinus was not assessed, and radiological diagnostics were not performed. It should be noted that the best imaging diagnostics to assess the healing of OAF would be MRI with contrast agents or some ultrasound techniques. However, none of the authors used these methods in their publications.

In the studies, second and third-generation platelet-derived preparations rich in growth factors were used. There were PRF and A-PRF. Only seven studies assessed the outcomes in groups of more than ten patients [5,7,13,14,19,22,23], and only three of them investigated the effect of platelet-derived concentrations rich in growth factors as the only treatment method on OAC healing. The use of platelet-derived preparations rich in growth factors as only methods was associated with a complete healing success rate [7,13,22]. The remaining studies had study groups smaller than ten patients, which may indicate a low quality of scientific evidence. Future studies should involve larger patient groups, with a preoperative assessment of the maxillary sinus condition using magnetic resonance imaging, computed tomography, cone beam computed tomography, ultrasonography, or endoscopy, using a single technique for closing the communications/fistulas or comparing platelet-derived preparations rich in growth factors with regional flaps. Thanks to this, the standards of procedures will be unified, and the treatments can become minimally invasive, with fewer complications. This is the future of treatment in oral and maxillofacial surgery.

## 5. Conclusions

Platelet-derived concentrations rich in growth factors can be used as the only method or support in the combined method (for example, platelet-derived preparations rich in growth factors and local flaps) in the treatment of oroantral communications or fistulas. When OAC is smaller than 5 mm, platelet-derived preparations rich in growth factors can be used as a single treatment technique. Most authors also reported that in addition to successful healing, after OAC using platelet-derived preparations rich in growth factors, there was a reduction in post-procedure complications such as pain and swelling. The use of blood products to close OAC may be an effective therapeutic alternative but requires evidence in a larger study group. Currently, the level of scientific evidence is insufficient. There is a need for future well-designed case-control or cohort studies and randomized controlled trials with the required level of evidence.

## Figures and Tables

**Table 1:** The studies included in the qualitative analysis and extracted data.

No	Study	Country	Patient Characteristics		Aim	OAC or OAF	OAC/OAF Diagnostics	Localization	Type of Platelet-Derived Preparations Rich in Growth Factors	Grown Factors Preparation	PRF Clot, Core or Membrane Using	Methods of Closure	Observation Period	Methods of Assessing the Success of Treatment	Level of OAC or OAF Closure Success	Additional Features	Conclusions
			Number	Age													
1	Śmieszek-Wilczewska et al. (2024) [13]	Poland	11 patients treated with using PRF	-	Compare the effectiveness of PRF and the conventional method of OAC repair techniques	OAC	Clinical and radiological examination (CBCT)	Not specified	A-PRF	-	Clots	PRF clots were put into the OAC defect and fixed on mucosal margins with sutures	14 days	Clinical and radiological assessment (CBCT)	Complete wound healing	-	OACs treated with A-PRF resulting in fewer complications and pain
2	Hunger et al. (2023) [14]	Austria	50	47.0 ± 14.9	The success of OAC closure after treatment with PRF clots or a BAF	OAC	Clinical and radiological examination (CBCT)	5 PM (1 case), 6 M (10 cases), 7 M (6 cases), 8 M (3 cases)	PRF	1300 rpm for 8 min at 210 G	Clots	2 PRF clots were inserted into the prepared sinus space, then full-thickness BAF was advanced into the palatal side	21 days	Clinical assessment	90%	BAF succeed rate 90%	The use of PRF is a successful method for the closure of OAC and is associated with lowered pain levels, a promising healing pattern and a good clinical soft tissue outcome. The defect size is decisive for an outcome
3	Bilginaylar (2019) [5]	Turkey	21 (with PRF)	No data	Compare the clinical outcomes of BAF to PRF for the closure of OAC	OAC	Clinical and radiological examination (CBCT)	No data	PRF	3000 rpm for 10 min	Clots	PRF, BFP and BAF	3 weeks	Clinical and radiological (X-ray) assessment	Patients were treated without any serious complications (alveolitis, infection, side effects, or the formation of OAF).	-	BAF and PRF are both useful for the closure of OACs. PRF application decreased pain and swelling compared to BAF
4	Bilginaylar (2018) [7]	Turkey	21 (with PRF)	No data	PRF clots were used for the flap-free closure of OAC after maxillary molar extraction	OAC	Clinical examination	No data	PRF	3000 rpm for 10 min	Clots	Only PRF	3 weeks	Clinical and radiological (X-ray) assessment	Patients were treated without any serious complications (alveolitis, infection, side effects, or the formation of OAF)	-	PRF is an alternative technique for the closure of OAC
5	Demetoglu et al. (2018) [19]	Turkey	21	No data	To evaluate the treatment of OACs with PRF	OAC	Clinical examination	5 PM (1 case), 6 M (14 cases), 7 M (4 cases); 8 M (1 case),	PRF	1500 rpm for 8 min	Membrane	PRF membrane, BFP, BAF	2 months	Clinical and radiological (X-ray) assessment	Complete wound healing	-	PRF can be used in the treatment of OACs with a diameter of 5 mm or less with a low risk of complications
6	Gülşen (2016) [22]	Turkey	20	No data	PRF clots for the flap-free treatment of OAC after tooth extraction	OAC	No data	No data	PRF	400 G for 10 min	Clots	PRF and sutures	3 weeks	Clinical assessment	Healing and epithelization of oral mucosa	-	OAC closure was achieved after PRF application
7	Kapustecki et al. (2016) [23]	Poland	20	No data	The usefulness of autogenous bone graft and PRF in normal bone regeneration in the site of OAC	OAC	Clinical and radiological examination (OPG, CBCT, Waters X-rays)	No data	Autogenous PRF	2700 rpm for 12 min	PRF membrane	Bone graft, PRF and BAF	3 to 6 months	Clinical and radiological (dental X-ray, Waters X-ray and CBCT) assessment	In 18 patients healing was complete	Measurement alveolar width values	Autogenous bone graft and PRF can be another way for the single-stage closure of a OAC and alveolar bone augmentation

BAF—buccal advanced flap; BFP—buccal fat pad; CBCT—cone-beam computed tomography; OAC—oroantral communication; OPG—orthopantomography; PRF—platelet-rich fibrin; 5 PM—second premolar; 6 M—first molar, 7 M—second molar, 8 M—wisdom tooth.

**Table 2:** Risk of bias using the Newcastle–Ottawa Scale for quality assessment.

No	Study	Sample Selection				Comparability		Exposure		Total
		Adequate Case Definition	Representativeness of the Cases	Selection of Control	Definition of Control	Comparability of Cases	Controls Based on the Analysis	Ascertainment of Exposure	Non-Response Rate	
1	Śmieszek-Wilczewska et al. (2024) [13]	★	★	★	★	★	★	★	★	**8**
2	Hunger et al. (2023) [14]	★	★	★	★	★	★	★	★	**8**
3	Bilginaylar (2019) [5]	★	★	-	★	★	-	★	★	**6**
4	Bilginaylar (2018) [7]	★	-	-	-	★	-	-	-	**2**
5	Demetoglu et al. (2018) [19]	★	★	-	-	-	-	★	-	**3**
6	Gülşen (2016) [22]	★	★	-	-	-	-	★	-	**3**
7	Kapustecki et al. (2016) [23]	★	-	-	-	★	-	★	-	**3**

Star (★) = item present, - = item not present.

## Data Availability

The data presented in this study are available upon request from the corresponding author. The data are not publicly available due to privacy restrictions.

## Supplementary Materials

The following supporting information can be downloaded at https://www.mdpi.com/article/10.3390/ijms252111507/s1.

## Abbreviations

ASCs	adipose-derived stem cells
AFG	autologous fibrin glue
A-PRF	advanced platelet-rich fibrin
BAF	buccal advanced flap
BFP	buccal fat pad
BMP-2	bone morphogenic factor 2
BMP-7	bone morphogenic factor 7
BMSCs	bone marrow stem cells
CBCT	cone beam computed tomography
CT	computed tomography
CGF	concentrated growth factors
EMMA	endoscopic middle meatal antrostomy
FGF	fibroblast growth factor
GBR	guided bone regeneration
HGF	hepatocyte growth factors
I-PRF	injectable platelet-rich fibrin
IGF	insulin-like growth factor
IL-1β	interleukin IL-1β
IL-4	interleukin IL-4
IL-6	interleukin IL-6
KT	kinesio tapes
L-PRF	leukocyte-platelet-rich fibrin
MRI	magnetic resonance imaging
MRONJ	medication-related osteonecrosis of the jaw
OAC	oroantral communication
OAF	oroantral fistula
OPG	orthopantomography
PD-EGF	platelet-derived epidermal growth factor
PDGF	platelet-derived growth factor
PDGF-AB	platelet-derived growth factor (built from chaim A and B)
PRF	platelet-rich fibrin
PRGF	plasma-rich in growth factors
PRP	platelet-rich plasma
T-PRF	titanium platelet-rich fibrin
TGF	transforming growth factor
TGFβ-1	transforming growth factor β-1
TNFα	tumor necrosis factor α
TSP-1	thrombospondin-1
VEGF	vascular-endothelial growth factor
4 PM	first premolar
5 PM	second premolar
6 M	first molar
7 M	second molar
8 M	wisdom tooth
