# Supplementary material for: Treatment of Oroantral Communication and Fistulas with the Use of Blood-Derived Platelet-Rich Preparations Rich in Growth Factors: A Systematic Review
Source: Int J Mol Sci. 2024 Oct 26;25(21):11507. doi: 10.3390/ijms252111507 (PMC11546229; doi:10.3390/ijms252111507)
Supplement: Supplementary file 1 [file ijms-25-11507-s001.zip › ijms-3244980-supplementary.pdf]

Table S1. Studies on OAC and OAF with study groups of less than ten cases.

No	Study	Country	Patient characteristic		Aim	OAC or OAF	OAC/OAF diagnostics	Localiza tion	Type of prepa- rations rich in growth factors	Grown factors prepa- ration	PRF clot, core or membrane using	Methods of closure	Observation period	Methods of as- sessing the success of treatment	Level of OAC or OAF closure success	Additional features	Conclusions
			Number	Age													
1	Elgabarty et al (2024) [12]	Egypt	9	Mean age 38	Use of endoscopic middle meatal antrostomy (EMMA) technique with the application of a PRF for OAF closure and maxillary sinusitis treatment	OAF	Clinical and radiological examination (CBCT)	Not speci- fied	PRF	3000 rpm for 10 min	Membranes	Endoscopic cleaning of the maxillary si- nus, fistula excision, PRF membrane placement, horizon- tal suture placement, intraoral acrylic splint adjustment to cover the PRF mem- branes	24 weeks	Radiological as- sessment (com- puted tomogra- phy)	Excellent	-	One-stage EMMA with the application of PRF membranes and acrylic splint is an alternative technique for OAF closure and maxillary sinusitis treatment that is associated with a lower incidence of complications
2	Adamska et al (2024) [1]	Poland	1	28	The role of A-PRF in the treatment of odontogenic maxillary sinusitis and immediate closure of extensive OAC	OAC	Clinical and radiological examination (CBCT)	7M	A-PRF	1500 rpm for 14 min	Clots	A-PRF clot and BAF	10 months	Clinical and radio- logical assessment (CBCT)	Complete wound healing	Treatment of maxil- lary sinusi- tis	In addition to providing OAC and supporting its healing, A-PRF may also influence the treatment of chronic sinusitis
3	Jung et al. (2023) [15]	Korea	2	47 and 48 (mean 47.5)	A "double-barrier technique" using PRF for chronic OAF closure	OAF	Clinical and radiological examination (OPG, CBCT)	6M and no data	PRF	3000 rpm for 15 min	Membranes	PRF clots were in- serted into the socket, then GBR membrane and BAF was advanced into the palatal side	3 months	Clinical and radio- logical assessment (CBCT)	Without com- plications	-	The use of a PRF membrane in a double-barrier technique can give good results in soft-tissue healing and easy closure of chronic OAF
4	Pal et al. (2022) [16]	India	1	22	Use of flapless double membrane closure of OAC with PRF and GTR membranes	OAC	Clinical and radiological examination (CBCT)	6M left side	PRF	3000 rpm for 12 min	Membranes	PRF, BFP and BAF	3 months	Clinical and radio- logical assessment (CBCT)	Thin cortical bone in CBCT	-	The sandwich technique demonstrates good clinical regeneration
5	Esen et al. (2021) [4]	Turkey	7	50-73 (mean 64.7)	Treatment of MRONJ resulting in OAF by performing sequestrectomy , PRF and BFP	OAF	Clinical examination	MRONJ	PRF	3000 rpm for 10 min	Membrane	PRF and BFP	18 months	Clinical assessment	Complete wound healing	MRONJ	OAF caused by MRONJ were successfully treated with a combined an antibiotic regimen, sequestrectomy, PRF and BFP
6	Chen et al. (2019) [6]	Taiwan	1	53	PRF and surgical approach for treatment OAF	OAF	Clinical and radiological examination (OPG, CBCT)	6M	PRF	No data	No data	PRF membranes and clot	10 months	Clinical and radio- logical (X-ray and CBCT) assessment	The wound healing was un- eventful	Osteoradi- onecrosis	PRF combined with a surgical approach might be useful for treatment of OAF and

No	Study	Country	Patient characteristic		Aim	OAC or OAF	OAC/OAF diagnostics	Localization	Type of preparations rich in growth factors	Grown factors preparation	PRF clot, core or membrane using	Methods of closure	Observation period	Methods of assessing the success of treatment	Level of OAC or OAF closure success	Additional features	Conclusions
			Number	Age													
					and osteoradionecrosis												osteoradionecrosis
7	Pandikanda et al. (2019) [17]	India	3	No data	Closure of OAC using the PRF and collagen	OAC	Clinical examination	No data	PRF	3000 rpm for 10 min	Membranes and clots	Collagen membrane, PRF clot, PRF membrane, free tension flap	2 months	Clinical assessment	The wounds healing were good.	-	The use of PRF membrane and mixture of PRF and collagen sandwiched between 2 PRF membranes allows immediate closure of OAC
8	Al-Juboori et al. (2018) [18]	Iraq	1	32	The combination of resolvable collagen membranes and PRF clots to close a chronic OAF	OAF	Clinical and radiological examination (OPG)	6M	A-PRF	1500 rpm for 14 min	Clots	Only PRF	6 weeks	Clinical assessment	Complete wound healing	-	Gingival tissue hypertrophy observed after 6 weeks of healing
9	George (2018) [20]	USA	1	77	BAF, BFP pedicle flap, and L-PRF use to close OAF in a chronic smoker	OAF	Clinical and radiological examination (CBCT)	4PM	L-PRF	No data	Membranes	Only PRF	9 days	Clinical assessment	No postoperative complications	-	Triple-layered technique is a to close a chronic OAF
10	Assad et al. (2017) [21]	Yemen	2	29 and 44, mean 36.5	Describe two cases of OAC with closed using PRF as a membrane and a clot	OAC	Clinical and radiological examination	6M	PRF	3000 rpm for 10 min	Clots and membranes	PRF and sutures	8 weeks	Clinical and radiological (X-ray) assessment	Complete success (no inflammatory reaction, signs, or symptoms of maxillary sinusitis)	-	PRF is a biomaterial for the closure of OAC
11	DePoi et al. (2007) [24]	USA	1	-	The use of PRP in the management of an OAF occurring as a complication of lateral wall sinus augmentation	OAF	No data	Sinus	PRP	-	-	PRP and flap	6 years	Clinical and histological assessment	-	-	-

BAF – buccal advanced flap; BFP – buccal fat pad; CBCT – cone-beam computed tomography; EMMA – endoscopic middle meatal antrostomy; GBR – guided bone regeneration; MRONJ – medication-related osteonecrosis of the jaw; OAC – oroantral communication; OAF – oroantral fistula; OPG – orthopantomography; PRF – platelet-rich fibrin; PRP – platelet-rich plasma; 4PM – first premolar; 5PM – second premolar; 6M – first molar, 7M – second molar, 8M – wisdom tooth
